# A Comparative Study on Effects of Three Butyric Acid-Producing Additives on the Growth Performance, Non-specific Immunity, and Intestinal Microbiota of the Sea Cucumber *Apostichopus japonicus*

**DOI:** 10.1155/2024/6973951

**Published:** 2024-02-17

**Authors:** Longzhen Liu, Cong Wei, Yongmei Li, Mingyang Wang, Yuze Mao, Xiangli Tian

**Affiliations:** ^1^Key Laboratory of Mariculture, Ministry of Education, Ocean University of China, Qingdao 266003, China; ^2^Yellow Sea Fisheries Research Institute, Chinese Academy of Fishery Sciences, Qingdao 266071, China

## Abstract

The providers of butyric acid, *Clostridium butyricum* (CB), sodium butyrate (SB), and tributyrin (TB), have been extensively studied as aquafeed additives in recent years. However, no comparative study has been reported on the probiotic effects of CB, SB, and TB as feed additives on sea cucumber (*Apostichopus japonicus*). A 63-day feeding trial was performed to assess the effects of dietary live cells of *C. butyricum* (CB group, the basal diet supplemented with 1% CB), sodium butyrate (SB group, the basal diet supplemented with 1% SB), and tributyrin (TB group, the basal diet supplemented with 1% TB) on the growth, non-specific immunity, and intestinal microbiota of *A. japonicus* with a basal diet group as the control. Results indicated that all three additives considerably increased *A. japonicus* growth, with dietary CB having the optimal growth-promoting effect. Of the seven non-specific enzyme parameters measured in coelomocytes of *A. japonicus* (i.e., the activities of phagocytosis, respiratory burst, superoxide dismutase, alkaline phosphatase, acid phosphatase, catalase, and lysozyme), dietary CB, SB, and TB considerably increased the activities of six, five, and six of them, respectively. The immune genes (*Aj*-p105, *Aj*-p50, *Aj*-rel, and *Aj*-lys) expression in the mid-intestine tissues of *A. japonicus* was significantly increased by all three additives. The CB group had the highest expression of all four genes. Additionally, the relative expression of *Aj*-p105, *Aj*-p50, and *Aj*-lys genes was significantly up-regulated in the three additive groups after stimulation with inactivated *Vibrio splendidus*. Dietary CB enhanced the intestinal microbial diversity and richness in *A. japonicus* while dietary TB decreased them. Meanwhile, dietary CB, SB, and TB significantly enhanced the abundance of Firmicutes, *unclassified_f_Rhodobacteraceae*, and Proteobacteria, respectively, while dietary CB and SB reduced the abundance of *Vibrio*. Dietary CB and SB improved the stability of microbial ecosystem in the intestine of *A. japonicus*. In contrast, dietary TB appeared to have a negative effect on the stability of intestinal microbial ecosystem. All three additives improved the intestinal microbial functions associated with energy production and immunity regulation pathways, which may contribute directly to growth promotion and non-specific immunity enhancement in *A. japonicus*. Collectively, in terms of enhancing growth and non-specific immunity, as well as improving intestinal microbiota, dietary live cells of *C. butyricum* exhibited the most effective effects in *A. japonicus*.

## 1. Introduction

The sea cucumber *Apostichopus japonicus* aquaculture industry plays crucial roles in supplying high-quality protein and promoting the development of the mariculture economy in China [1], with an area exceeding 250,000 hm^2^ and a production exceeding 248,000 t in 2022 [2]. However, slow growth, disease outbreaks, and antibiotic misuse remain significant constraints and ongoing challenges to the health development of *A. japonicus* farming [3, 4]. Providing high-quality feed can supply sufficient nutrients to animals, enhance their immunity and disease resistance, and decrease the likelihood of disease [5]. The development of feed additives that are beneficial, such as growth promoters and immunostimulants, has become increasingly critical and necessary for the health of aquaculture production [5, 6].

Butyric acid (BA, C_4_H_8_O_2_) is a short-chain fatty acid produced by bacterial fermentation of dietary carbohydrates. It has attracted increasing focus for its beneficial effects on the intestine [7]. BA not only provides direct energy to intestinal cells but also enhances the absorption of nutrients by the intestinal mucosa. Several studies have shown that BA can inhibit enteric pathogens, promote health, and enhance the growth and immunity of the aquatic animals [7], such as hybrid catfish *Clarias macrocephalus* × *C. gariepinus* [8], African catfish *Clarias gariepinus*, Nile tilapia *Oreochromis niloticus* [9], and Barramundi *Lates calcarifer* [10].

In the aquaculture industry, BA salts are commonly used as additives in aquafeeds instead of BA due to their stability and palatability [11]. Sodium butyrate (SB, C_4_H_7_O_2_Na) is a stable salt of BA. It can be included directly in animal feed without requiring a coating step [12]. Previous researches have proven that SB can maintain intestinal health, as well as improve the growth and immunity in aquatic animals, such as turbot *Scophthalmus maximus* L. [13], Nile tilapia *O. niloticus* [14], largemouth bass *Micropterus salmoides* [15], and Pacific white shrimp *Litopenaeus vannamei* [16]. Tributyrin (TB, C_15_H_26_O_6_) is a precursor of BA composed of three BA lipid molecules and a glycerol backbone. It is more stable and produces more BA in the intestine compared to other forms of BA [11, 17]. It has been shown in several studies that TB has beneficial effects on intestinal morphology and microbiota, lipid metabolism, and the growth in aquatic animals, including yellow drum *Nibea albiflora* [18], large yellow croaker *Larimichthys crocea* [17], and black sea bream *Acanthopagrus schlegelii* [19]. Furthermore, *Clostridium butyricum* (CB), a butyric acid-producing probiotic, can provide nutrients to intestinal cells and promote the repair of intestinal epithelial cells [20]. Researches have demonstrated that CB can enhance the growth, feed conversion efficiency, and bacterial infection resistance in aquatic animals [21].

The SB and TB are different BA forms, and CB is the most important butyric acid-producing probiotic. It is demonstrated that the addition of CB, SB, and TB into feed resulted in increased growth performance and immunity and improved intestinal microbiota structure and function in fish and crustaceans [22, 23]. However, limited research has been carried out on using CB, SB, and TB as feed additives for *A. japonicus*. To address this research gap, this study assessed the effects of CB, SB, and TB on the growth, non-specific immunity in coelomocytes, immune genes expression levels in intestinal tissues, and intestinal microbiota for *A. japonicus*. A comprehensive comparison of the beneficial effects of the three additives on *A. japonicus* was performed. The results will provide a scientific foundation for the application of CB and butyrate as feed additives or as an alternative to antibiotics in *A. japonicus* farming practices.

## 2. Materials and Methods

### 2.1. Experimental Animals and Experimental Diets

Healthy *A. japonicus* were obtained from Yantai Anyuan Aquatic Products Co., Ltd. (Yantai, China). The *A. japonicus* were acclimatized for 15 days at a temperature of 18 ± 1°C, salinity of 29 ± 1‰, pH of 8.0 ± 0.2, and dissolved oxygen of 8.0 ± 0.2 mg/L and fed a basal diet.

The CB and TB additives (>98% purity) were supplied by Qingdao GBW Group (Qingdao, China). The CB contained spray-dried spores with live cells at a concentration of 5 × 10^9^ colony forming units (CFU) per gram. The SB (>98% purity) was obtained from the ADDEASY Group (Weifang, China). The basal diet (containing 70% sea mud, 15% sargassum powder, and 15% commercial feed) was prepared using our previous method [24] and served as the control diet (CO group).  Table 1 presents the proximate composition of the commercial feed. The basal diets were mixed separately with CB, SB, and TB additives at a concentration of 1% (*w/w*) to create the experimental diets, designated the CB, SB, and TB groups, respectively (Table 2). The powdered diets were mixed with sterile seawater to create a paste-like consistency. All diets were freshly prepared daily and fed to the *A. japonicus* within an hour.

### 2.2. Experimental Design and Management

After acclimatization, the *A. japonicus* were starved for 24 hr before the feeding trial. A total of 200 *A. japonicus* (6.49 ± 0.07 g, mean ± SE) were randomized to 20 aquaria (55 cm × 30 cm × 35 cm), with 10 individuals per aquarium. The 20 aquaria were randomly assigned into four feeding groups: CO (control), CB, SB, and TB groups, and five replicates were presented in each group. During the feeding trial period, which lasted 63 days, the *A. japonicus* were fed once daily at 3% of body weight at 6:00 PM. The rearing conditions during the feeding trial were identical to those during the acclimation period. The aquaria were cleaned by removing the feces and feed residues with a siphon tube and one-third of the water was replaced before feeding daily.

### 2.3. Sampling and Non-specific Immune Parameters Analyses

Following the feeding trial, the *A. japonicus* were subjected to a 24-hr period of starvation. The growth performance of *A. japonicus* was calculated for each group. Three *A. japonicus* were randomly selected in each aquarium to collect the coelomic fluid, mid-intestine tissues, and intestinal contents under aseptic conditions. For each sample type, triplicate samples in each aquarium were combined into one sample. The mid-intestine tissues and intestinal contents were freeze-clamped using liquid nitrogen and preserved at −80°C prior to RNA and DNA extraction, respectively. The coelomic fluid was added into an equal volume of anticoagulant (0.48 M NaCl, 0.019 M KCl, 0.02 M EGTA, 0.068 M Tris-HCl, pH 7.6) [25], and the coelomocytes number was counted in per milliliter. A fraction of fresh coelomic fluid was utilized for assays measuring phagocytosis and respiratory burst activities. The cell lysate supernatant was prepared from the remaining fraction of coelomic fluid for the assays of superoxide dismutase (SOD), catalase (CAT), acid phosphatase (ACP), alkaline phosphatase (AKP), and lysozyme (LZM) activities, following our previously described methods [24].

### 2.4. Inactivated *Vibrio Splendidus* Stimulation Assay

Purified *V. splendidus* strain was supplied by the Laboratory of Aquaculture Ecology, Ocean University of China. The preparation of inactivated *V. splendidus* suspension (2.57 × 10^8^ CFU/mL) was prepared, as described previously [24].

After sampling, the remaining 30 *A. japonicus* in each group were randomized to three aquaria, with 10 individuals in each aquarium. The stimulation assay involved injecting 100 *μ*L of inactivated *V. splendidus* suspension into *A. japonicus* coelomic cavity. The rearing conditions during the stimulation assay were identical to those during the feeding trial. At 24 and 72 hr post-injection, mid-intestine tissues of three *A. japonicus* in each aquarium were collected for immune genes expression assay.

### 2.5. Relative Quantification of Immune Genes

Total RNA extraction was performed with Trizol Reagent (Ambion, USA) from the mid-intestine tissues of *A. japonicus* in both the feeding trial and inactivated *V. splendidus* stimulation assay. The RNA was verified for integrity and purity, then converted to cDNA. The relative expression of *Aj*-p105, *Aj*-p50, *Aj*-rel, and *Aj*-lys genes was determined using quantitative real-time PCR, as our previously established procedures [24]. The equation 2^−*ΔΔ*CT^ was used to calculate the target gene expression. A detailed list of primers is available in Table 3.

### 2.6. Intestinal Microbiota Analysis

The PowerFecal DNA Isolation Kit (Mobio, USA) was utilized to harvest total DNA from intestinal contents of *A. japonicus*. After verifying the quality of DNA, the primers 338F/806R were applied to amplify the V3-V4 region of the 16S rDNA according to our previously established procedure [24] and sequenced on an Illumina MiSeq platform.

The raw sequences underwent quality filtering using QIIME (v. 1.9.1) to obtain the valid sequences. The operational taxonomic units (OTUs) clustering was processed on an identity threshold of 97% valid sequences. The resulting OTU sequences were annotated with Silva reference database (v. 138). Rarefaction curves and a Venn diagram were generated. The Chao1, ACE, Shannon, Simpson, and Coverage indices were calculated, and their statistical significance was determined across groups in R software (v. 4.3.1) and RStudio (v. 2023.09.0-463). Non-metric multidimensional scaling (NMDS) was used to evaluate the similarity of intestinal microbial community. Linear discriminant analysis effect size (LEfSe) was performed with non-parametric Kruskal–Wallis test to analyze OTUs differences across groups (https://www.bic.ac.cn/ImageGP/). The statistical significance level was set at *P* < 0.05, and a cutoff LDA score of 3.0 was applied. In addition, the Pearson correlation among the growth parameters and the changed intestinal microbiota was performed using the R software (v. 4.3.1) and RStudio (v. 2023.09.0-463).

Intestinal microbial networks were built in Cytoscape (v. 3.10.1) using Co-occurrence network inference. Only OTUs detected in more than 3/5 of samples were retained for network construction. Four algorithms, namely Pearson correlation, Spearman correlation, Bray–Curtis dissimilarity, and Kullback–Leibler dissimilarity, were employed to construct networks. The threshold for edge selection was set to 500 top and bottom. The final networks were obtained through a procedure of randomization and bootstrap. The *P* value merge method chosen was the Brown method, and the Benjamini–Hochberg procedure was employed for multiple test correction. Edge scores were calculated for 100 iterations, and unstable edges (with a *P* value threshold of 0.05) were filtered out during the randomization and bootstrap step. Using the R (v. 4.3.1) package igraph and RStudio (v. 2023.09.0-463), 1,000 random networks were constructed. The topology property parameters of the networks were calculated. And the statistical significance of the average clustering coefficient, average path distance, and modularity between empirical and random networks in each group, and then between the control and other groups were conducted using a one-sample *t*-test and a *t*-test, respectively. The networks were visualized using Gephi (v. 0.10).

Based on the KEGG database, the functions of the intestinal microbiota were inferred using PICRUSt2 (v. 2.4.1). Statistical comparisons of functional pathways were performed across the control and other groups in the STAMP software (v. 2.1.3) using two-sided Welch's *t*-tests, with significant differences considered at *P* < 0.05.

### 2.7. Calculations and Statistical Analysis

(1) $$\begin{array}{c} \text{Specific growth rate}\;\left(\text{SGR},\%/\text{day}\right)=\left(\text{Ln}W_{\text{e}}-\text{ln}W_{\text{s}}\right)\times 100/63, \end{array}$$

(2) $$\begin{array}{c} \text{Weight gain rate}\;\left(\text{WGR},\%\right)=\left(W_{\text{e}}-W_{\text{s}}\right)/W_{\text{s}}\times 100, \end{array}$$

(3) $$\begin{array}{c} \text{Survival rate }\left(\text{SR},\%\right)=N_{\text{e}}\times 100/N_{\text{s}}, \end{array}$$

where *W*_e_ and *W*_s_ are the mean final and mean initial weight, respectively; *N*_e_ and *N*_s_ are the final and initial number of *A. japonicus*, respectively.

The results were presented as mean ± SE (standard error of the mean). Significant differences (*P* < 0.05) across groups were determined by one-way analysis of variance with Duncan's multiple range test in R software (v. 4.3.1) and RStudio (v. 2023.09.0-463).

## 3. Results

### 3.1. Growth Performance

All three additives considerably elevated the SGR of the *A. japonicus (P*  < 0.05). The SGR was markedly higher in the CB and SB groups than in the TB group (*P* < 0.05). Dietary CB and SB considerably improved the WGR of the *A. japonicus (P*  < 0.05), whereas the dietary TB did not affect WGR considerably (*P* > 0.05). Furthermore, the WGR in the CB group was markedly higher compared to the TB group (*P* < 0.05). None of the three additives markedly affected the SR of the *A. japonicus (P*  > 0.05) (Table 4).

### 3.2. Phagocytosis and Respiratory Burst Activity

**Figure 1:**
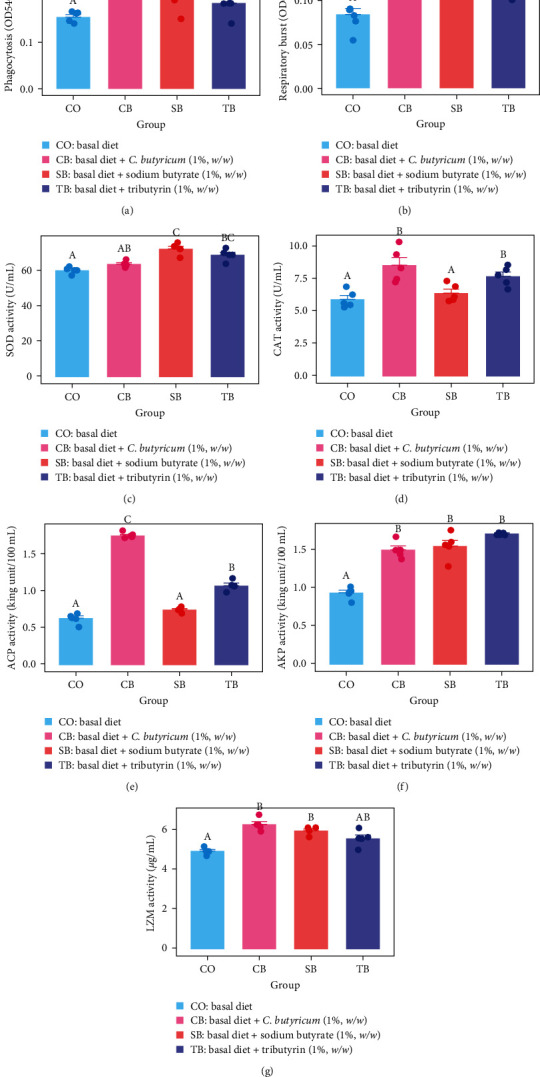
(a–g) Non-specific enzyme activities in coelomocytes of *A. japonicus* fed with four diets. Values (mean ± SE) with completely different superscripts are significantly different (*P* < 0.05). CO (control), basal diet; CB, basal diet + *C. butyricum* (1%, *w/w*); SB, basal diet + sodium butyrate (1%, *w/w*); TB, basal diet + tributyrin (1%, *w/w*).

All three additives considerably elevated the phagocytic and respiratory burst activities in the coelomocytes (*P* < 0.05). The CB group showed markedly higher phagocytic activity compared to the SB and TB groups (*P* < 0.05). No significant differences were detected in respiratory burst activity across the three additive groups (*P* > 0.05) (Figures 1(a) and 1(b)).

### 3.3. Non-specific Immune Enzymes Activities

Dietary SB and TB considerably elevated SOD activity (*P* < 0.05). Moreover, SOD activity was markedly higher in the SB group than in the CB group (*P* < 0.05) (Figure 1(c)).

The CAT and ACP activities were markedly higher in the CB and TB groups compared to the CO and SB groups (*P* < 0.05). Furthermore, ACP activity was markedly higher in the CB group than in the TB group (*P* < 0.05) (Figures 1(d) and 1(e)).

All three additives considerably improved AKP activity, and dietary CB and SB considerably improved LZM activity (*P* < 0.05) (Figures 1(f) and 1(g)).

### 3.4. Immune Genes Expression

**Figure 2:**
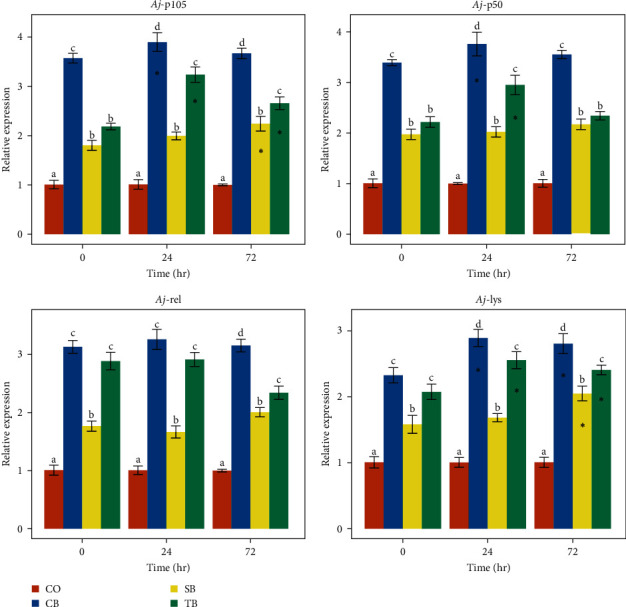
Effect of three additives on the relative expression of immune genes in *A. japonicus* mid-intestine tissues. Values (mean ± SE) in each time point with completely different superscripts are significantly different (*P* < 0.05). The asterisks ( ^*∗*^) indicated that the relative expression of the target gene at that time point differed significantly from that at time point 0 (*P* < 0.05). The time points of 0, 24, and 72 hr represented the prior to the injection of inactivated *V. splendidus*, followed by 24 and 72 hr after the injection. CO (control), basal diet; CB, basal diet + *C. butyricum* (1%, *w/w*); SB, basal diet + sodium butyrate (1%, *w/w*); TB, basal diet + tributyrin (1%, *w/w*).

After a 63-day feeding trial, all three additives considerably increased the immune genes expression in the mid-intestine tissues (*P* < 0.05). Among them, the CB group showed markedly higher *Aj*-p105 and *Aj*-p50 genes expression than the SB and TB groups (*P* < 0.05). The CB and TB groups exhibited markedly higher *Aj*-rel and *Aj*-lys genes expression than the SB group (*P* < 0.05) (Figure 2).

After 24 hr of stimulation with inactivated *V. splendidus*, the expression of *Aj*-p105, *Aj*-p50, and *Aj*-lys genes was considerably up-regulated in the CB and TB groups (*P* < 0.05). Meanwhile, after 72 hr of stimulation, the expression of *Aj*-p105 gene in the SB and TB groups and *Aj*-lys gene in all three additive groups was considerably up-regulated (*P* < 0.05) (Figure 2).

### 3.5. Intestinal Microbiota Analysis

#### 3.5.1. Microbial Diversity

**Figure 3:**
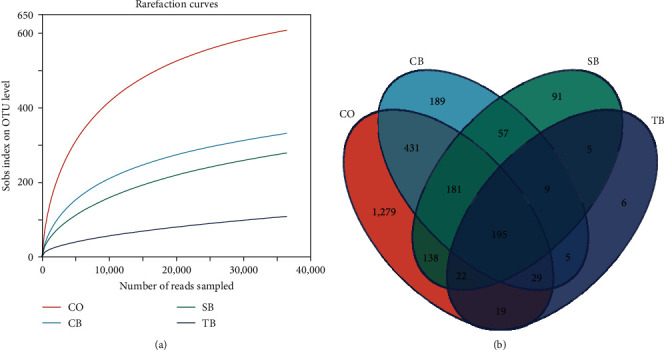
Rarefaction curves (a) and Venn diagram (b) analysis for *A. japonicus* intestinal microbiota. CO (control), basal diet; CB, basal diet + *C. butyricum* (1%, *w/w*); SB, basal diet + sodium butyrate (1%, *w/w*); TB, basal diet + tributyrin (1%, *w/w*).

**Figure 4:**
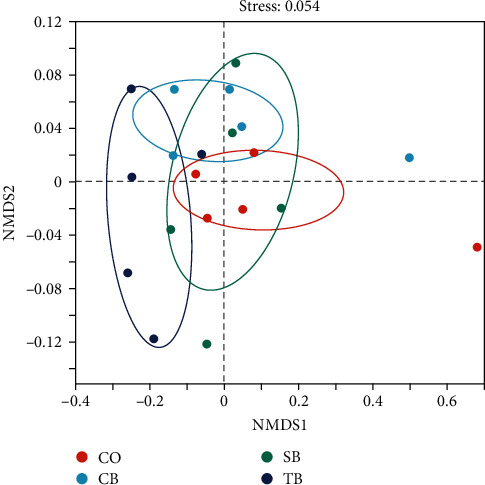
NMDS analysis for the *A. japonicus* intestinal microbiota. CO (control), basal diet; CB, basal diet + *C. butyricum* (1%, *w/w*); SB, basal diet + sodium butyrate (1%, *w/w*); TB, basal diet + tributyrin (1%, *w/w*).

The rarefaction curves approximately tended to a plateau above 36,449 reads in 20 samples, indicating that sufficient sequencing depth was achieved (Figure 3(a)). Venn diagram revealed that the CO, CB, SB, and TB groups had 1,279, 189, 91, and 6 characteristics of OTUs, respectively (Figure 3(b)). Compared to the CO group, the Chao1, Shannon, and Simpson in the CB group were markedly increased, while the ACE in the SB group and Chao1, ACE, and Shannon in the TB group were dramatically reduced (*P* < 0.05) (Table 5). The NMDS analysis revealed significant differences in intestinal microbial community structures among the CB, TB, and CO groups (Figure 4).

#### 3.5.2. Microbial Composition

**Figure 5:**
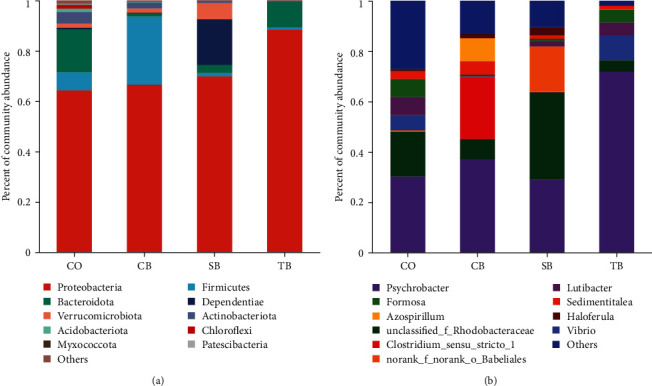
Effect of three additives on intestinal microbial community composition of *A. japonicus* at the phylum (a) and genus (b) levels. CO (control), basal diet; CB, basal diet + *C. butyricum* (1%, *w/w*); SB, basal diet + sodium butyrate (1%, *w/w*); TB, basal diet + tributyrin (1%, *w/w*).

The *A. japonicus* intestine was found to contain major bacterial phyla, including Proteobacteria, Firmicutes, Bacteroidota, Dependentiae, and Verrucomicrobiota. The TB (88.44%) and CB (27.16%) groups showed a noteworthy rise in the relative abundance of Proteobacteria and Firmicutes, respectively (*P* < 0.05). The relative abundance of Bacteroidota was considerably lower in the CB (1.15%), SB (3.23%), and TB (10.25%) groups (*P* < 0.05). Additionally, the relative abundance of Dependentiae and Verrucomicrobiota was markedly enriched in the SB (18.07%, 6.51%) group, while it was markedly reduced in the TB (0.03%, 0.16%) group (*P* < 0.05) (Figure 5(a)).

The *A. japonicus* intestine was found to contain major bacterial genera, including *norank_f_norank_o_Babeliales, Clostridium_sensu_stricto_1, Psychrobacter, unclassified_f_Rhodobacteraceae*, and *Lutibacter*. The relative abundance of *Psychrobacter* in the TB (71.94%) group, *unclassified_f_Rhodobacteraceae* (34.77%) and *norank_f_norank_o_Babeliales* (18.02%) in the SB group, and *Clostridium_sensu_stricto_1* in the CB (24.51%) group increased significantly (*P* < 0.05). Additionally, the relative abundance of *Vibrio* was considerably reduced in the CB (0.18%) and SB (0.04%) groups (*P* < 0.05) (Figure 5(b)).

**Figure 6:**
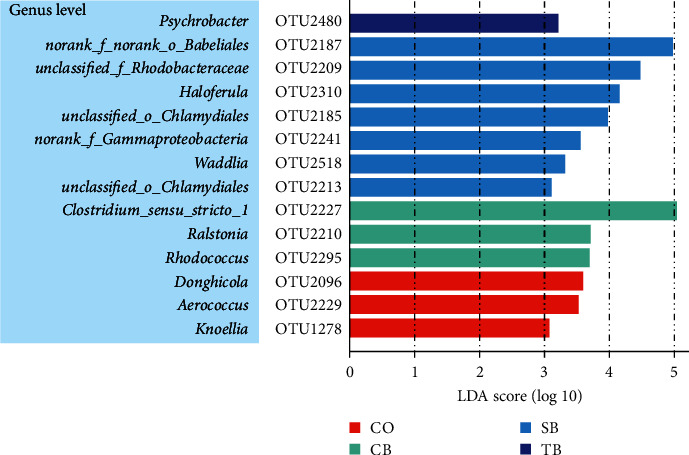
Differential abundance analysis using LEfSe identified the OTUs that were most significantly different between each group. CO (control), basal diet; CB, basal diet + *C. butyricum* (1%, *w/w*); SB, basal diet + sodium butyrate (1%, *w/w*); TB, basal diet + tributyrin (1%, *w/w*).

There were three, three, seven, and one OTUs with significantly different abundances in the CO, CB, SB, and TB groups, respectively, identified by LEfSe analysis (LDA score > 3.0). Among them, three OTUs in the CO group belonging to *Knoellia*, *Aerococcus*, and *Donghicola*. Three OTUs in the CB group belonging to *Clostridium_sensu_stricto_1*, *Rhodococcus*, and *Ralstonia*. Seven OTUs in the SB group belonging to *norank_f_norank_o_Babeliales, unclassified_o_Chlamydiales, Waddlia, norank_f_Gammaproteobacteria, Haloferula*, and *unclassified_f_Rhodobacteraceae*. One OTU in the TB group belongs to *Psychrobacter* (Figure 6).

#### 3.5.3. Correlation between the Growth Parameters and the Changes of Intestinal Microbiota

**Figure 7:**
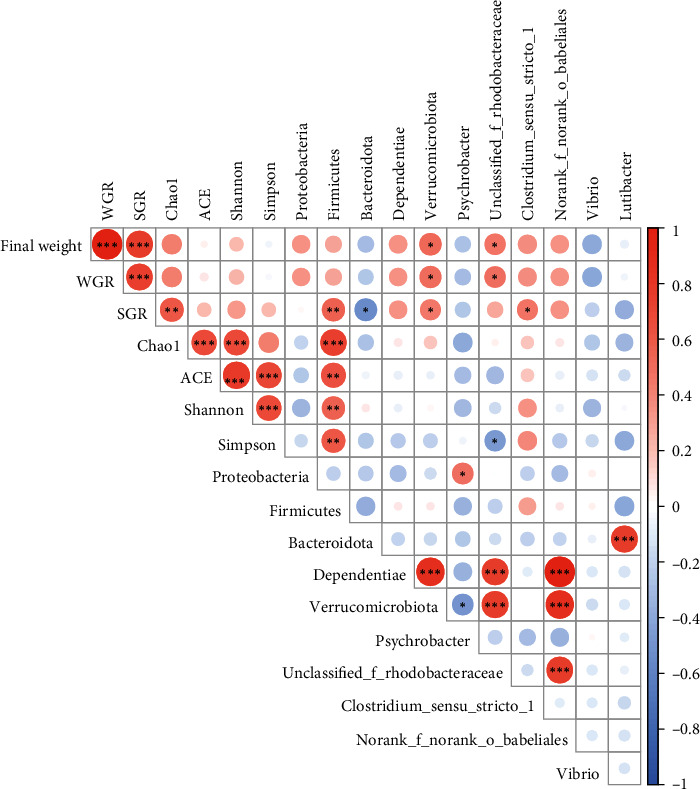
Pearson correlation among the growth parameters and the changes of intestinal microbiota ( ^*∗*^*P* < 0.05;  ^*∗∗*^*P* < 0.01;  ^*∗∗∗*^*P* < 0.001). The strength of correlation is indicated by the color intensity and circle size. Red indicates a positive correlation, while blue indicates a negative correlation.

To evaluate the correlation among the growth parameters, bacterial community alpha diversity indices, and major bacterial abundance, Pearson correlation was performed. The result showed that the final weight and WGR were significantly positively correlated with the abundance of Verrucomicrobiota and *unclassified_f_Rhodobacteraceae*. The SGR was significantly positively correlated with Chao1 index and abundance of Firmicutes, Verrucomicrobiota, and *Clostridium_sensu_stricto_1*, while significantly negatively correlated with the abundance of Bacteroidota (Figure 7).

#### 3.5.4. Microbial Ecological Network

**Figure 8:**
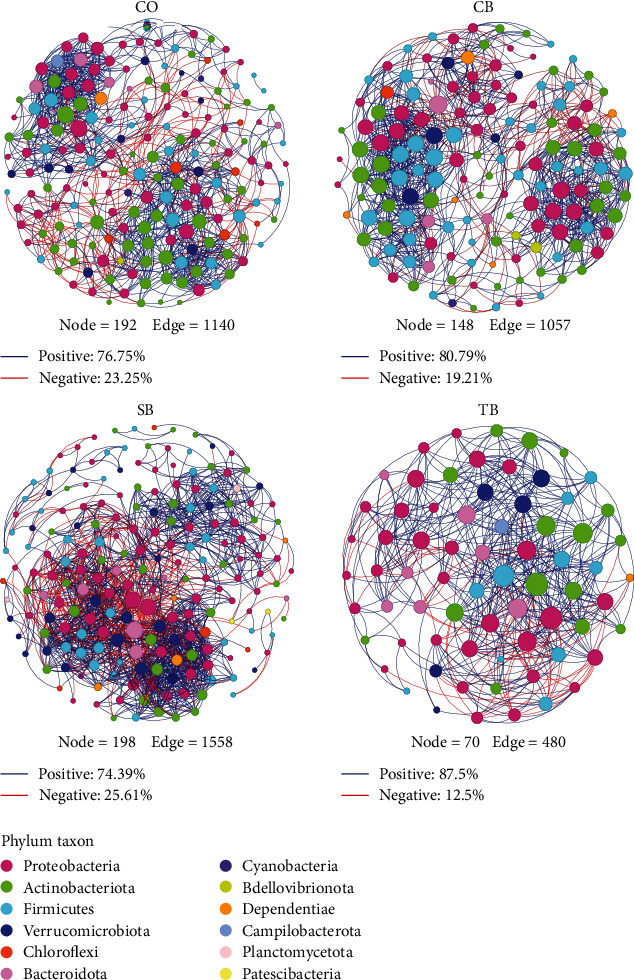
Intestinal microbial ecological networks of *A. japonicus*. The nodes, which were also known as OTUs, were color-coded to indicate different phyla. Negative interactions were represented by red links, while positive interactions were represented by blue links. CO (control), basal diet; CB, basal diet + *C. butyricum* (1%, *w/w*); SB, basal diet + sodium butyrate (1%, *w/w*); TB, basal diet + tributyrin (1%, *w/w*).

Co-occurrence network analysis was built to examine the effects of three additives on the microbial ecosystem in *A. japonicus* intestine. The result revealed that the microbial ecological networks in the CO, CB, SB, and TB groups comprised of 192 nodes and 1,140 edges, 148 nodes and 1,057 edges, 198 nodes and 1,558 edges, and 70 nodes and 480 edges, respectively (Table 6). Notably, the SB group network exhibited the highest node and edge counts. The average clustering coefficient, average path distance, and modularity in each group's empirical network and their corresponding random network significantly differed (*P* < 0.001). All three additives elevated the density and average degree of the networks. The TB and SB group networks had the highest values of density and average degree, respectively. The average clustering coefficient, average path distance, and modularity differed significantly between the CO group and three additive group networks (*P* < 0.001). The CB group network had the highest values of average clustering coefficient and modularity, while the SB group network had the highest value of average path distance (Table 6 and Figure 8).

#### 3.5.5. Microbial Function Prediction

**Figure 9:**
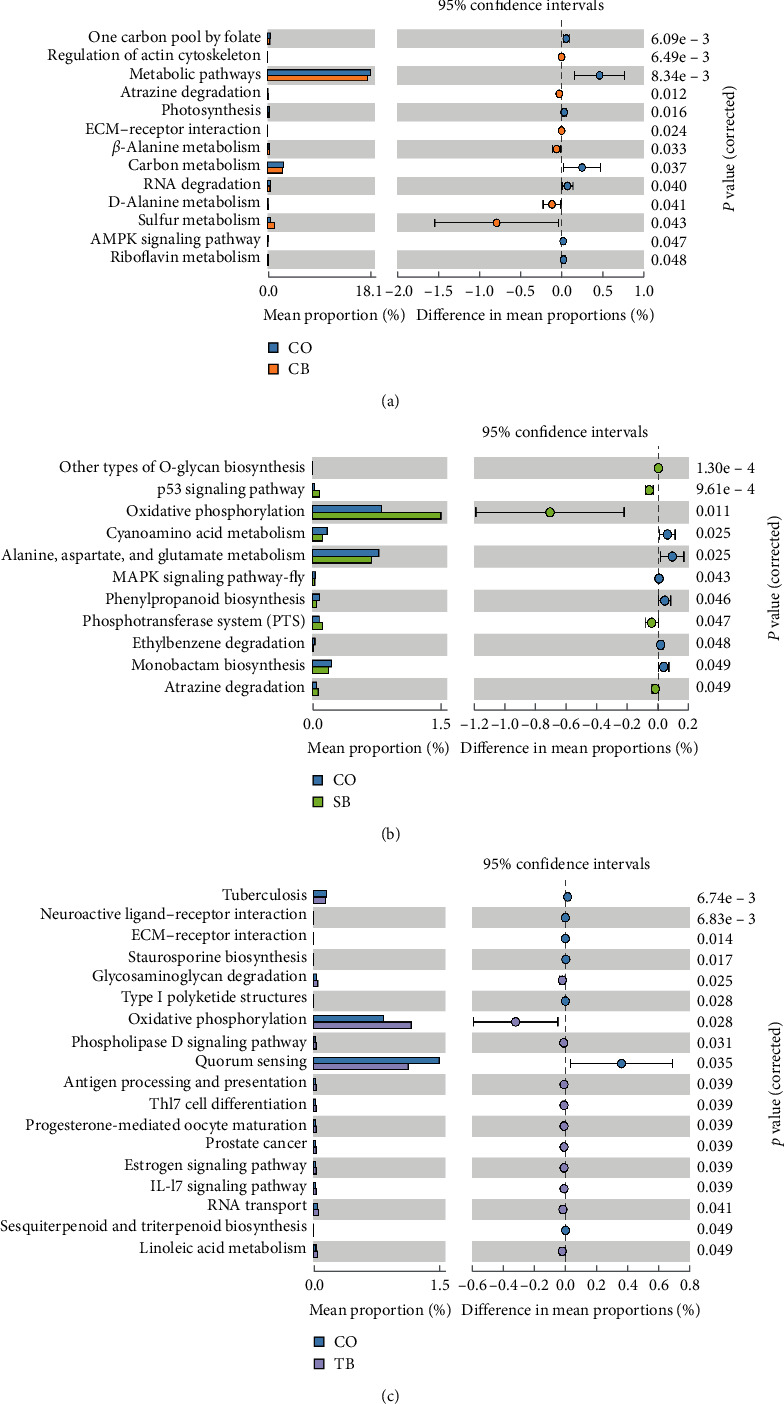
Effect of three additives on the intestinal microbial functions in *A. japonicus*. The KEGG functional pathways (level 3) of significant differences (*P* < 0.05) between the control (CO group) and the other groups were identified: (a) CO and CB groups; (b) CO and SB groups; (c) CO and TB groups. CO (control), basal diet; CB, basal diet + *C. butyricum* (1%, *w/w*); SB, basal diet + sodium butyrate (1%, *w/w*); TB, basal diet + tributyrin (1%, *w/w*).

The analysis of intestinal microbial function prediction revealed that dietary CB considerably enhanced six KEGG level 3 functional pathways, namely regulation of actin cytoskeleton, atrazine degradation, ECM–receptor interaction, *β*-alanine metabolism, D-alanine metabolism, and sulfur metabolism. Additionally, the CB group showed a significant attenuation in seven functional pathways, namely one carbon pool by folate, metabolic pathways, photosynthesis, carbon metabolism, RNA degradation, AMPK signaling pathway, and riboflavin metabolism (Figure 9(a)).

Dietary SB considerably enhanced five functional pathways, namely other types of O-glycan biosynthesis, oxidative phosphorylation, phosphotransferase system (PTS), atrazine degradation, and p53 signaling pathway. Additionally, the SB group showed significant attenuation in six functional pathways, namely cyanoamino acid metabolism, MAPK signaling pathway-fly, phenylpropanoid biosynthesis, ethylbenzene degradation, monobactam biosynthesis, and “alanine, aspartate, and glutamate metabolism” (Figure 9(b)).

Dietary TB considerably enhanced 11 functional pathways, namely antigen processing and presentation, linoleic acid metabolism, phospholipase D signaling pathway, glycosaminoglycan degradation, oxidative phosphorylation, progesterone-mediated oocyte maturation, RNA transport, prostate cancer, estrogen signaling pathway, Thl7 cell differentiation, and IL-l7 signaling pathway. The TB group showed significant attenuation in seven functional pathways, namely tuberculosis, neuroactive ligand–receptor interaction, ECM-receptor interaction, staurosporine biosynthesis, type I polyketide structures, quorum sensing, and sesquiterpenoid and triterpenoid biosynthesis (Figure 9(c)).

## 4. Discussion

Butyric acid (BA) and its salts, including sodium butyrate (SB) and tributyrin (TB), have gained more attention as growth promoters, immunostimulants, and for their antioxidant properties in aquatic animals [7, 22]. Meanwhile, the use of *C. butyricum* (CB) as a feed additive has increased due to its capacity to produce BA. According to this study, the growth of *A. japonicus* increased notably with the dietary supplementation of CB, SB, and TB. And the CB group exhibited the highest SGR and WGR, indicating that CB had the greatest growth-promoting for *A. japonicus*.

The *A. japonicus* possess both cellular and humoral immunity as part of their innate immune system. Coelomocytes are a crucial element of the innate immune system in *A. japonicus*. They can eliminate pathogenic microorganisms through phagocytosis, respiratory burst, and synthesis of humoral protective factors and non-specific immune enzymes [26, 27]. The non-specific immune capacity of *A. japonicus* can be assessed through the activities of phagocytosis, respiratory burst, and several non-specific immune enzyme activities, such as SOD, CAT, ACP, AKP, and LZM in coelomocytes. In this study, both the dietary CB and TB considerably elevated six of the non-specific immunity metrics (except SOD and LZM in the CB and TB groups, respectively), and dietary SB significantly increased five of the non-specific immunity metrics (except CAT and ACP). The CB group presented the highest activities of phagocytosis, CAT, ACP, and LZM, while SB group presented the highest activities of respiratory burst and SOD. The TB group presented the highest AKP activity. The *Aj*-p105, *Aj*-p50, *Aj*-rel, and *Aj*-lys genes regulate immune responses in *A. japonicus*, and the expression levels of these genes can also reflect the non-specific immune capacity of *A. japonicus* [28–30]. All three additives greatly enhanced these four immune genes expression in intestinal tissues of *A. japonicus* in this study. Dietary CB demonstrated the strongest ability to enhance expression of immune genes, followed by the dietary TB. Furthermore, the *Aj*-p105, *Aj*-p50, and *Aj*-lys genes expression was greatly up-regulated in all three additive groups following stimulation with inactivated *V. splendidus*. Above mentioned results indicated that dietary CB, SB, and TB can enhanced the non-specific immunity of *A. japonicus*. The dietary containing CB achieved the best immune-enhancing effect for *A. japonicus*.

The research showed that the CB, SB, and TB can alter the intestinal microbiota of aquatic animals by producing BA, such as juvenile yellow drum *N. albiflora* [31], Pacific white shrimp *L. vannamei* [32, 33], largemouth bass *M. salmoides* [15], and mirror carp *Cyprinus carpio* [34]. Dietary addition of CB considerably increased the intestinal microbial diversity and richness, while the addition of TB dramatically decreased them in *A. japonicus*. The decrease in diversity and richness in the TB group may be attributed to its higher dosage. TB consists of a glycerol backbone and three BA lipid molecules. This form of BA is more stable and produces greater amounts of BA in *A. japonicus* intestine compared to other forms [21]. Additionally, TB has a long half-life and slow metabolism, which results in the slow and prolonged release of BA [35]. The pH in the intestine of *A. japonicus* decreased continuously as BA accumulated. An overly acidic intestinal environment may inhibit the growth and reproduction of acid-intolerant microorganisms, resulting in lower diversity and richness in the intestine of *A. japonicus*. Therefore, the addition of 1% TB (*w/w*) may be too high for *A. japonicus*. However, further research is needed to explain these phenomena. The analysis of NMDS indicated that the intestinal microbial structure was altered by three additives, and there were clear separations among the control, CB, and TB groups especially. Meanwhile, the LEfSe analysis identified 14 significantly abundant OTUs in four groups, with seven of them were present in the TB group. Moreover, the greatly increased abundance in *unclassified_f_Rhodobacteraceae*, Firmicutes, and Proteobacteria was present in the SB, CB, and TB groups, respectively. The dietary CB and SB significantly decreased the *Vibrio* abundance. Furthermore, the Pearson correlation analysis revealed that the abundance of Verrucomicrobiota and *unclassified_f_Rhodobacteraceae* had a positive correlation with WGR, and the abundance of Firmicutes and Verrucomicrobiota had a positive correlation with SGR. Previous studies have shown that Firmicutes break down carbohydrates and degrade plant cell wall components to produce short-chain fatty acids, which provide nutrients for the host [36]. Furthermore, some Firmicutes have the ability to boost host immunity by upregulating immune genes expression [37]. *Rhodobacteraceae* possesses complex metabolic pathways and participates in a variety of ecological functions [38, 39]. Most members of *Rhodobacteraceae* can synthesize vitamin B_12_ necessary for *A. japonicus* growth. Additionally, some members of the *Rhodobacteraceae* exhibit potentially inhibitory effects on pathogens through secondary metabolites [40, 41]. Proteobacteria have been identified as being strongly implicated in the degradation of a variety of complex compounds [42]. Verrucomicrobiota members are crucial in the degradation of polysaccharides [43]. *Vibrio* is generally considered primary opportunistic pathogen in causing disease and death in aquatic animals [44]. The results suggested that the dietary CB, SB, and TB enhanced the abundance of potentially beneficial bacteria and reduced the abundance of opportunistic pathogens in *A. japonicus* intestine. This may directly contribute to the enhancement of growth and non-specific immunity for *A. japonicus*.

The bacterial species interact in a complex ecological network in *A. japonicus* intestine, which regulates the balance and stability of the intestinal microbial ecosystem [45]. A network with more nodes and edges generally indicates more diverse the relationships between species. Meanwhile, the network that has a higher density, average degree, average clustering coefficient, and modularity indicates that it is more complex and stable [45–47]. The SB and TB groups had the largest (the most nodes and edges) and smallest (the least nodes and edges) networks in the present study, respectively. Additionally, all three additives increased the network density and average degree. The modularity in the CB group network, as well as average clustering coefficient in the CB and SB group networks considerably higher compared to the control. Conversely, these values were significantly reduced in the TB group. The above results implied that dietary CB and SB enhanced the stability of microbial ecosystem in *A. japonicus* intestine. Nevertheless, the dietary TB appeared to have a negative effect on the stability of intestinal microbial ecosystem. This may be associated with the reductions in the intestinal microbial diversity and richness resulting from a 1% TB-supplemented diet [48].

Intestinal microbiota is essential in regulating growth and immune response in *A. japonicus* [49]. In this study, the *β*-alanine metabolism, D-alanine metabolism, and regulation of actin cytoskeleton pathway were considerably enriched in the CB group than in the control. Enhanced functional pathways of *β*-alanine metabolism and D-alanine metabolism may allow more nutrients and energy to be available to the *A. japonicus*, thus promoting its growth [50]. The regulation of actin cytoskeleton pathway is critical to the activation of immune cells, which mediate the immune response [51]. The PTS pathway and the oxidative phosphorylation pathway were considerably enriched in the SB group than in the control. Research has shown that PTS pathway enables bacteria to efficiently utilize glucose in challenging environments [52]. Oxidative phosphorylation is the primary source of ATP production [53, 54]. The linoleic acid metabolism, glycosaminoglycan degradation, and oxidative phosphorylation pathway were considerably enriched in the TB group than in the control. Enhancement of the linoleic acid metabolism pathway facilitates the promotion of lipid utilization in *A. japonicus* [55]. Reports suggested an important role for the glycosaminoglycan degradation pathway in the modulation of growth factor signaling [50]. The enhancement of the glycosaminoglycan degradation pathway probably provides more energy for the *A. japonicus* growth [49]. These findings revealed that the dietary CB, SB, and TB can improve intestinal microbial functions, leading to improved *A. japonicus* growth and non-specific immunity.

## 5. Conclusion

Dietary *C. butyricum*, sodium butyrate, and tributyrin can significantly increase the growth performance and non-specific immunity of *A. japonicus*. Of these, dietary *C. butyricum* had the most significant positive effect. Additionally, dietary *C. butyricum* elevated the intestinal microbial diversity and richness in *A. japonicus*, while dietary tributyrin decreased them. The supplemented diets of *C. butyricum* and sodium butyrate significantly reduced the abundance of the opportunistic pathogen *Vibrio* and improved the stability of the microbial ecosystem in the intestine of *A. japonicus*. However, the addition tributyrin appeared to have a negative effect on the stability of the intestinal microbial ecosystem. Three additives improved the intestinal microbial functions associated with energy production and immunity regulation pathways, which may contribute to improved growth and non-specific immunity in *A. japonicus*. In a thorough comparison of growth enhancement, non-specific immunity, and improvement of intestinal microbiota among three additives, *C. butyricum* showed the best effects for *A. japonicus*.

## Figures and Tables

**Table 1:** Proximate composition of the commercial feed.

Nutrients	Percentage (%)
Crude protein	20.0
Crude lipid	2.5
Crude fiber	10.0
Crude ash	36.0
Moisture	4.0

**Table 2:** The experimental groups for comparison of three additives as dietary supplements in *A. japonicus*.

Group	Diets
CO (control)	Basal diet
CB	Basal diet contained 1% (*w/w*) *Clostridium butyricum*
SB	Basal diet contained 1% (*w/w*) sodium butyrate
TB	Basal diet contained 1% (*w/w*) tributyrin

CO (control), basal diet; CB, basal diet + *C. butyricum* (1%, *w/w*); SB, basal diet + sodium butyrate (1%, *w/w*); TB, basal diet + tributyrin (1%, *w/w*).

**Table 3:** Detailed information of primers used in this study.

Gene	Primer name	Primer sequence (5′−3′)	Product size (bp)	Source or GenBank
*β*-Actin	Actin-F	TTATGCTCTTCCTCACGCTATCC	104	AB510191
	Actin-R	TTGTGGTAAAGGTGTAGCCTCTCTC		

*Aj*-p105	p105-F	GCAACACACCCCTCCATCTT	148	[25]
	p105-R	TCTTCTTCGCTAACGTCACACC		

*Aj*-p50	p50-F	TCCTATCGGTCTGAATCTTCCAA	133	[25]
	p50-R	TTTCTTCCCTTTCTGGCTATGTTC		

*Aj*-rel	Rel-F	TGAAGGTGGTATGCGTCTGG	132	JF828765
	Rel-R	TTGGGCTGCTCGGTTATG		

*Aj*-lys	Lys-F	AGGGAGGTAGTCTGGATGGA	138	EF036468
	Lys-R	GCGCAAAATCCTCACAGGTA		

**Table 4:** Growth performance of *A. japonicus* fed with four diets for 63 days.

Index	Group			
	CB	SB	TB	CO
Initial weight (g)	6.50 ± 0.05^a^	6.52 ± 0.03^a^	6.49 ± 0.03^a^	6.45 ± 0.03^a^
Final weight (g)	11.90 ± 0.41^b^	11.63 ± 0.75^b^	10.01 ± 0.23^a^	9.29 ± 0.58^a^
SGR (%/day)	1.03 ± 0.04^c^	0.95 ± 0.03^c^	0.76 ± 0.04^b^	0.65 ± 0.01^a^
WGR (%)	83.04 ± 5.99^c^	78.45 ± 11.41^bc^	54.19 ± 3.32^ab^	44.28 ± 9.50^a^
SR (%)	100	100	100	100

Values (mean ± SE) in the same row with completely different superscripts are significantly different (*P* < 0.05). CO (control), basal diet; CB, basal diet + *C. butyricum* (1%, *w/w*); SB, basal diet + sodium butyrate (1%, *w/w*); TB, basal diet + tributyrin (1%, *w/w*). SGR, specific growth rate; WGR, weight gain rate; SR, survival rate.

**Table 5:** Alpha diversity indices of intestinal microbiota.

Group	Richness		Diversity		Coverage
	Chao1	ACE	Shannon	Simpson	
CB	578.60 ± 56.50^c^	532.35 ± 63.50^b^	2.60 ± 0.17^c^	0.79 ± 0.00^b^	0.997
SB	470.60 ± 10.15^bc^	337.17 ± 35.45^a^	1.79 ± 0.14^b^	0.58 ± 0.05^a^	0.997
TB	288.64 ± 28.10^a^	277.78 ± 29.22^a^	1.14 ± 0.13^a^	0.57 ± 0.04^a^	0.998
CO	410.66 ± 36.61^b^	462.54 ± 21.34^b^	2.09 ± 0.16^b^	0.68 ± 0.03^a^	0.997

Values (mean ± SE) in the same column with completely different superscripts are significantly different (*P* < 0.05). CO (control), basal diet; CB, basal diet + *C. butyricum* (1%, *w/w*); SB, basal diet + sodium butyrate (1%, *w/w*); TB, basal diet + tributyrin (1%, *w/w*).

**Table 6:** Topological properties of the empirical and their random networks.

Group	Empirical networks							Random networks		
	Nodes	Edges	Network density	Average degree	Average clustering coefficient	Average path distance	Modularity	Average clustering coefficient	Average path distance	Modularity
CB	148	1,057	0.097	14.28	0.60^a,b^	3.19^a,b^	0.59^a,b^	0.13 ± 0.02	2.24 ± 0.03	0.02 ± 0.001
SB	198	1,558	0.080	15.74	0.57^a,b^	3.52^a,b^	0.48^a,b^	0.16 ± 0.01	2.38 ± 0.04	0.02 ± 0.001
TB	70	480	0.199	13.71	0.52^a,b^	2.17^a,b^	0.36^a,b^	0.21 ± 0.01	1.98 ± 0.01	0.02 ± 0.001
CO	192	1,140	0.062	11.88	0.56^a^	3.43^a^	0.57^a^	0.10 ± 0.02	2.49 ± 0.03	0.02 ± 0.001

The letter “a” indicates a significant difference between the empirical and their random networks at a significance level of *P* < 0.001. The letter “b” indicates a significant difference in values within the same column between the control and other groups at a significance level of *P* < 0.001. CO (control), basal diet; CB, basal diet + *C. butyricum* (1%, *w/w*); SB, basal diet + sodium butyrate (1%, *w/w*); TB, basal diet + tributyrin (1%, *w/w*).

## Data Availability

Raw sequencing data was submitted to NCBI SRA (PRJNA1053772).
